# An Offenders-Offenses Shared Component Spatial Model for Identifying Shared and Specific Hotspots of Offenders and Offenses: A Case Study of Juvenile Delinquents and Violent Crimes in the Greater Toronto Area

**DOI:** 10.1007/s10940-022-09562-9

**Published:** 2022-11-05

**Authors:** Jane Law, Abu Yousuf Md Abdullah

**Affiliations:** 1 School of Planning, University of Waterloo, 200 University Avenue West, Waterloo, ON N2L 3G1 Canada; 2 School of Public Health Sciences, University of Waterloo, Waterloo, ON Canada

**Keywords:** Bayesian, Shared component spatial modeling, Offenders-offenses shared patterns, Juvenile delinquents, Violent crime

## Abstract

**Objectives:**

We attempted to apply the Bayesian shared component spatial modeling (SCSM) for the identification of hotspots from two (offenders and offenses) instead of one (offenders or offenses) variables and developed three risk surfaces for (1) common or shared by both offenders and offenses; (2) specific to offenders, and (3) specific to offenses.

**Methods:**

We applied SCSM to examine the joint spatial distributions of juvenile delinquents (offenders) and violent crime (offenses) in the York Region of the Greater Toronto Area at the dissemination area level. The spatial autocorrelation, overdispersion, and latent covariates were adjusted by spatially structured and unstructured random effect terms in the model. We mapped the posterior means of the estimated shared and specific risks for identifying the three risk surfaces and types of hotspots.

**Results:**

Results suggest that about 50% and 25% of the relative risks of juvenile delinquents and violent crimes, respectively, could be explained by the shared component of offenders and offenses. The spatially structured terms attributed to 48% and 24% of total variations of the delinquents and violent crimes, respectively. Contrastingly, the unstructured random covariates influenced 3% of total variations of the juvenile delinquents and 51% for violent crimes.

**Conclusions:**

The Bayesian SCSM presented in this study identifies shared and specific hotspots of juvenile delinquents and violent crime. The method can be applied to other kinds of offenders and offenses and provide new insights into the clusters of high risks that are due to both offenders and offenses or due to offenders or offenses only.

## Introduction

Crime is a complex multidimensional process, where the overall risk of a crime can be viewed as a product of the risks originating from the offenders, the offenses, or even both. Why some crimes are recurrent at some specific sites and what characteristics of these places drive offenders to criminal activities remains an area of active research (Wortley and Townsley 2016). Numerous studies have confirmed the presence of a spatial structure of crime and have recognized the phenomena as a construct of the latent processes or the social, cultural, and environmental factors (Law et al. 2015, 2014; Jennings et al. 2011; Griffiths and Chavez 2004). Crime is non-random and non-uniform across space and society, as evidenced by localized clusters or hotspots of crime (Johnson and Bowers 2004; Johnson et al. 1997). However, the complex interactions between the spatially structured and unstructured risk factors complicate the study of crime risk patterns, and advanced modeling techniques are required to estimate the crime-shared (by offenders and offenses) and crime-specific (offenders or offenses-specific) risks in an area (Law et al. 2015, 2014; Li et al. 2014). In this paper, we aim to introduce a different approach to study hotspots of crime that is based on offenders and offenses together (rather than separately), and a statistical model to identify shared and specific hotspots based on outcome data of offenders and offenses. Knowing these hotspots would provide insights for more precise targeting of offenders and/or offenses locations in law enforcement planning.

### Shared and Specific Hotspots: Theoretical Frameworks

Both offenders and offenses tend to cluster geographically and, thus, hotspots on crime maps. According to the *crime pattern theory*, offenders tend to commit crimes near their residences or neighborhoods (usually poorer and clustered), where they feel much more comfortable. Shared (at the same locations) hotspots of offenders and offenses are formed when offenders and victims both live at the location where the crime takes place, referred to as a *crime neighborhood triangle* in the mobility triangle (Normandeau 1968). Most studies of the mobility triangle, a typology of the spatial relations between the offender, victim, and criminal event have used the neighborhood as a spatial unit of analysis (Groff and McEwen 2007; Tita and Griffiths 2005). On the other hand, offenses-specific hotspots are found in neighborhoods with certain characteristics that attract crime, where the homes of the offenders and victims concerned are in the same neighborhood, but the offense occurs elsewhere (*offense mobility triangle*), or they may be from elsewhere in entirely different neighborhoods (*total mobility triangle*).

Hotspots of offenders and offenses may or may not overlap. We use "shared hotspots" or "offenders-offenses hotspots" to refer to hotspots of offenders and offenses (crime-shared or crime-general) that overlap (e.g., where both are frequent in poorer neighborhoods for domestic violent crime), and "specific hotspots" (crime-specific) to refer to hotspots that do not overlap. Specific hotspots can be offenders-specific (e.g., poorer neighborhoods that have many offenders but the offenders travel to commit property crime in richer neighborhoods), offenses-specific (e.g., richer neighborhoods have more property crime), or even victim-specific. We will focus on offenders and offenses (not victims) for this paper.

Crime research has studied hotspots, mostly specific, i.e., based on offenders or offenses. It is generally agreed that hotspots are formed due to the locational features of an area promoting the activities of offenders (Brantingham and Brantingham 2016). Studies have shown that public places such as shopping malls, parks, and transport stations, where a large number of people gather, act as "crime generators" or as catalysts for offenders to commit crimes (Bernasco 2010; Brantingham and Brantingham 1995). Similarly, some places such as poorly lit streets, bars, drug markets, and brothels act as "crime attractors" by encouraging offenders to commit an offense, particularly because the victims at these places are not well protected (Miller et al. 2016). What type of offense an offender will commit at these places is not clear but can somehow be understood using the *rational choice theory*. For example, violent crimes (VC) such as murder or physical assault usually require more than one person, and the frequency with which these crimes will occur will depend on the assistance received from other offenders (Miller et al. 2016; Andresen and Jenion 2010; Johnson et al. 2009). Thus, while the crime pattern theory shows that some areas could be offenses-specific hotspots due to their proximity to offenders, the rational choice theory suggests that some areas could also be offenders-specific hotspots because of the availability of motivated offenders with similar interests.

In addition to the ecological theories, the social theories shed essential light on the shared risks of the offenders and offenses. The *social disorganization theory* suggests that social cohesion will determine how well a society deters offenses (Sampson et al. 1997). A society with strong social bonding between the people is likely to offer greater protection against crimes than a society with low cohesion. Generally, people sharing similar beliefs and values connect well and help each other to protect themselves from crime; this might be in the form of discouraging vulnerable people from committing crimes or directly stopping offenders from committing offenses. However, factors such as racism, poverty, and economic inequality can disrupt social networks, leading to increased crime frequencies in an area (Eck et al. 2005). The offenders' perception of a society's cohesion will dictate where (near or away from their residences) and how often they will commit a crime and, thus, will determine whether an area will be a crime hotspot. A similar principle can be found in the *broken windows theory*, which suggests that once a society overlooks a small offense, the society's ability to subdue crimes is undermined, leading to a chain reaction where people become reluctant to deal with bigger transgressions in the future (Gau and Pratt 2010). This deviancy in dealing with small offenses creates a 'broken window,' allowing offenders to get away with bigger offenses. Thus, these two theories suggest that the strength of guardianship determines both the number of new offenders and offenses in a particular area.

Combining these four common theories discussed in environmental criminology, we see that "poor guardianship" in an area (social disorganization theory) gives the locational features (crime pattern theory) that motivate vulnerable individuals to commit crimes and get away without any repercussions (broken windows theory). The lack of guardianship thus increases the number of offenders, making it feasible to execute more severe crimes that require assistance from other offenders (rational choice theory), which in turn, again contributes to the locational features that attract more offenders (crime pattern theory). Hence, these theories are highly interconnected, suggesting that the relationship between offenders and offenses can only be adequately captured when the conjoint risks due to offenders and offenses are studied in tandem with the offenders- and offenses-specific risks of crime.

### Studying Hotspots of Offenders or/and Offenses: A Review of Past Methods

Hotspot analyses are often exclusively focused on offenders or offenses and generally consider these two as distinct entities (offenders or offenses-specific hotspots). In studies where both the offenders and offenses were studied together, risk estimation was conducted individually and later compared to obtain an idea of the shared risk (Bailey and Gatrell 1995). These objectives were achieved by past studies using any of the three different types of approaches: (i) the univariate and multivariate tests, (ii) multivariate modeling, or (iii) a combination of both tests and model-based approaches.

One example of the univariate test-based approach is the study conducted by Bowers et al. (2004) to identify offense-specific hotspots. They had modified the moving window technique, which is conventionally used for hotspot mapping, and used a recursive algorithm to generate a *risk intensity* surface for burglaries (the offense). First, weights were generated based on the location and time of the point events. The sum of the inverse weights was then used to calculate the risk intensity and identify the offense-specific hotspots of burglary (Bowers et al. 2004). Their objective was to introduce an event-based hotspot mapping as opposed to traditional area-based approaches. Consequently, they have used 10 × 10 and 50 × 50 m grids to summarize the risk intensity values and to generate the risk surfaces for hotspot identification. In addition to grids, past studies have also employed circles or ellipses to implement different univariate techniques for hotspot detection, such as K-means clustering, kernel density estimation, and Kulldorff's spatial or space–time scans (Khalid et al. 2018; Miller et al. 2016; Malleson and Andresen 2015; Kennedy et al. 2011; Yu et al. 2011; Bowers et al. 2004; Johnson and Bowers 2004).

The use of such univariate and shape-based techniques to study the areal pattern creates a number of problems. First, in most studies, both the selection of the bandwidth and grid sizes are based on guesswork or as per the needs of the study objectives (Bowers et al. 2004). This arbitrary selection is theoretical and may introduce the modifiable areal unit problem (MAUP). The MAUP, in turn, affects the intra-class correlation or the relative impact of the surrounding on the offenders and offenses, leading to unreliable estimates of the risk levels (Gerell 2017). Second, in hotspot modeling using the techniques mentioned earlier, event-based or point data are frequently used, where the 'small number problem' can become a concerning issue if proper statistical techniques are not employed to analyze the hotspots. Since most of the statistical validations involve testing whether the observed distribution of crime significantly differs from a randomized dataset of the same crime, the size of data used for counting the crimes can affect the sensitivity of the hypothesis tests (Johnson et al. 2009). Third, univariate test-based approaches do not allow offenders and offenses to be studied simultaneously. Therefore, these approaches are limited to studying offenders-specific and offenses-specific hotspots only. Hence, the proportion of variance in offenders or offenses-specific risks owing to the shared risks of offenders and offenses cannot be ascertained.

Multivariate test-based approaches include the use of principal component analysis (PCA), local autocorrelation measures, or hotspot detection techniques such as Geary's C and local Moran's *I* (Anselin 2019; Zhang and McCord 2014). For example, PCA can be used to group offenders or offenses into specific components to understand their colocation. The assumption is that the different types of data that fall into the same group or component should also have locational similarities or should co-locate in space (Shearmur and Coffey 2002). Pope and Song (2015) followed this principle and studied the colocation of multiple types of crimes using PCA (Pope and Song 2015). They used PCA to group different offenses into four major components or types of crime (contraband, violent, property, and theft). The component scores of each major crime type in each block of the study area were then used as proxy measures of risks and mapped to illustrate the hotspots.

There are three major problems associated with the use of non-spatial techniques, such as PCA, to identify hotspots. First, as PCA is heavily reliant on non-spatial attributes of the data and assumes that similarity in non-spatial attributes will indicate a similarity in spatial attributes, the spatial and non-spatial attributes influencing the observations of offenders or offenses cannot be considered as separate entities. Therefore, the spatial autocorrelation in the data can neither be estimated nor adjusted during risk mapping. Second, studying the colocation of offenders and offenses could be heavily complicated by the fact that offenders tend to get involved in multiple types of crimes. So, offenders may show a high correlation with multiple types of offenses, making the separability of major components extremely difficult. Third, component scores, which Pope and Song (2015) had used as proxy measures for risks of various types of crimes, would be difficult to interpret when the colocation of offenders and offenses are studied. This is because the component scores do not have any intuitive meaning. So, when the variables are non-identical, for example, offenders are persons and offenses are events, the component scores do not have any straightforward interpretations for risk measures.

Most importantly, the univariate and multivariate methods described above are based on statistical tests rather than models and have limited applicability compared to statistical model-based techniques. A statistical test simply checks whether there is enough evidence (from the data) to conclude that the distribution of the offenders or offenses is not random. In contrast, a statistical model can incorporate the relationship between one or more measured or unmeasured and spatial or non-spatial covariates during hotspot mapping (Robertson et al. 2010). The model then checks whether these relationships are due to random chances or have occurred as a result of the influence of underlying latent processes (Kleinbaum et al. 2013). In that way, statistical models can better approximate reality and consider the effect of multidimensional factors while analyzing hotspots.

However, it is also important to consider that not all statistical models are suitable in spatial contexts. For example, some conventional statistical models either do not adjust the spatial autocorrelation in the data or consider spatial effects as a nuisance and adjust them accordingly (Izenman et al. 2011; Fotheringham et al. 2003). For example, multivariate modeling techniques such as the co-clustering or biclustering technique can be used to study offenders and offenses together. Izenman et al. (2011) employed the plaid-biclustering technique to identify the hotspots of urban juvenile delinquents (JD) getting involved in recidivism. In a plaid-biclustering technique, columns and rows that cluster simultaneously in a matrix of data are detected (Izenman et al. 2011). The plaid-biclustering differs from conventional biclustering because it allows the use of non-continuous datasets and the overlapping of clusters (i.e., where the columns and rows with data can belong to more than one cluster type) (Shabalin et al. 2009). Using this method, they assigned the JD into specific groups, which in turn were defined by variables such as type of initial offense and background characteristics of offenders (Izenman et al. 2011). However, as the plaid-biclustering algorithm cannot consider the nature of the distribution of the data and mainly relies on non-spatial data to create the groups of delinquents, a separate spatial cluster analysis using Getis–Ord *G*_*i*_ statistic (Ord and Getis 1995; Getis and Ord 1992) was necessary to identify the hotspots of JD showing recidivism. The plaid-biclustering technique has similar drawbacks as mentioned earlier for PCA; it cannot account for the spatial autocorrelation in the data or how the neighbors influence the observations in an area.

Past studies have also explored the applicability of mixed methods to study offenders and offenses simultaneously in a single study. Mixed methods involve collecting and analyzing both qualitative and quantitative data to utilize the strengths of both methods. The quantitative part of the analysis can integrate either the multivariate test or model-based approaches to identify the hotspots. For example, Porter et al. (2019) developed hotspot maps of crime (drug and violence-related) in a high-crime neighborhood in Ohio, using calls to police, spatial video, and geonarrative methodology. The process involved locating *'perceived'* hotspots of offenses using the narratives from police officers and neighborhood residents. These officers and residents were selected through non-random sampling strategies. The perceived hotspots of offenders (the locations of high crime intensity) were then identified with the help of ex-offenders. These hotspots were later merged with the hotspots identified through the *'Call for Service'* data from local police departments. The intensity of crime or the risk estimation was conducted using a kernel density estimation of crime mentions (such as drug or violence) and a quadratic function was used to reduce the locational errors owing to the increase of distance from the center of the kernel (Porter et al. 2019). However, several problems exist with such mixed-method techniques. At first, the perceptual identification of hotspots could be heavily subjective. Indeed, the perceptual hotspots identified by the community, police officers, and ex-offenders had significant discrepancies with the crime mentions hotspots. Second, this type of technique is resource-intensive, as spatial video and geonarrative techniques might not be suitable for mapping hotspots in large areas. Moreover, the number of ex-offenders might be limited, which may result in selection bias during the identification of offenders-specific hotspots. Selection bias may also arise due to the non-random sampling of the geonarrative participants (such as the residents and police officers) and affect the mapping of offenses-specific hotspots. Therefore, unless offenders- and offenses-specific hotspots are accurately identified, the offenders-offenses hotspots cannot be mapped with reasonable precision.

### Shared Component Spatial Modeling: An Alternative Method to Identify Hotspots of Offenders and Offenses

As discussed in the literature review, the majority of previous research has focused on offenders-specific and offenses-specific hotspots. Few studies have analyzed these two types of hotspots together by considering them separately in one joint model. A study exploring the shared and specific hotspots of offenders and offenses in a single spatial model is missing.

Consequently, this study employed a shared component spatial modeling (SCSM) to identify offenders-offenses shared, offenders-specific and offenses-specific hotspots at a small-area scale. Assuming that the outcomes of offenders and offenses may share some common risk factors like socioeconomic or/and built environmental factors, the SCSM can jointly analyze the spatial variations (i.e., the maps) of offenses and offenders for identifying shared (e.g., due to common risk factors) and specific (e.g., due to uncommon risk factors) hotspots. Apart from SCSM, we have considered the use of multivariate conditional autoregressive modeling (MVCARM). The MVCARM is also used for analyzing two or more different but possibly correlated spatial outcomes, and correlations are estimated using the observed data in the model. Both SCSM and MVCARM can produce more reliable estimates for small areas where counts are often low because parameter estimation is done by borrowing strength across areas and amongst the correlated outcomes. However, unlike SCSM, MVCARM does not combine maps to show shared and specific hotspots, and thus, was not chosen for this study (MacNab 2010). For further comparison of the two modeling approaches, see MacNab (2010).

This study implemented the shared component spatial model in a Bayesian platform to analyze offenders and offenses jointly at a small-area level, which has several advantages. First, small-area analysis often involves small counts, referred to as the small-number problems, which, in turn, makes the trend and risk estimate unreliable (Law et al. 2014). A small-number problem can also arise due to detection bias and misclassification of geographic data (Law and Perlman 2018). In this regard, Bayesian models can 'borrow' information from neighboring areas and adjust for the small-number problems or any redundancies in data owing to the sampling, reporting, and classification artifacts (Law et al. 2006).

Second, overdispersion is a common phenomenon in count data. Overdispersion occurs when there is higher variance in the observed data than expected. The presence of overdispersion in a Poisson model (used to model count data) underestimates the Type I errors during the hypothesis tests and can also underestimate the standard errors of parameter estimates (Law et al. 2006). This problem has been tackled in a Bayesian model using two random terms representing the spatially structured (*s*_*ik*_) and unstructured (*u*_*ik*_) effects. These two random terms allow the model to capture the unmeasured spatially structured and unstructured covariates, which influence the relative risks of offenders and offenses. Furthermore, Bayesian models do not treat the spatial effects as a nuisance, as can be observed in cases of other statistical models (Izenman et al. 2011). This allows Bayesian models to estimate the extent to which spatial components of the risk dominate the overall risk in an area.

Third, and most importantly, SCSM enables joint analysis of offenders and offenses in one statistical model but as two separate outcomes that are analyzed together. It is an extension of the most frequently used Besag, York and Mollie model (BYM) (Besag et al. 1991) for Bayesian spatial modeling and has been found to perform better than separate analyses of individual outcomes by the BYM (Downing et al. 2008). Thus, the SCSM can filter the area-specific risks into three components: (1) shared by the offenders and offenses (crime-shared or offenders-offenses shared), (2) offenders-specific, and (3) offenses-specific.

Despite some major constraints, offenders and offenses-specific risks could also be modeled using conventional statistical models. However, the superiority of the Bayesian models stems from its ability to estimate the shared risk from the same (shared component of offenders and offenses) model from which it estimates the specific risks. The shared and specific risks in the model have a locally dependent prior probability structure that enables the identification of hotspots. Further strengths of the model will be explained in terms of the model components in the “Methods” section.

The “Methods” Section describes the study region, data, and the shared component model. The results of the analysis are presented in “Results” Section, and the relevant discussions highlighting the significant findings, strengths, and limitations of the study are included in “Discussion” Section. The “Conclusion” Section reports the conclusions with recommendations for future research.

## Methods

### Study Area and Data

Using the data from the York Regional Police Department (YRPD), Ontario, Canada, we applied and empirically tested the SCSM to differentiate offenders-offenses shared component from offenders-specific and offenses-specific components. The Regional Municipality of York, where we were able to acquire the crime data for this study, was our study region. York Region is one of the municipalities in Toronto, Ontario, Canada, and in 2006, the population of the city was 892,712, with 841,897 (94%) of the population living in the urban centers. The areal unit of analysis is the dissemination area (DA), which is the smallest geographic unit created to disseminate the census data. The DAs have a uniform standard population that generally varies between 400 and 800 persons and covers the entire territory of Canada (Statistics Canada 2018). A total of 1,128 DAs make up the York Region, each having an average population of 791 people with an average area of 1.90 km^2^.

The data available for our modeling of offenders and the offenses are juvenile delinquents (JD) and violent crimes (VC). The JD data provided covers not just VC but all crime, which is a data limitation and is discussed later in detail. We geocoded the addresses of offenders and offenses to geographic coordinates (x, y) using ArcGIS 10.0. The success rate of geocoding was 98%. The total counts of offenders or offenses in each DA were obtained using a point-in-polygon method, which involved counting and summing the incidents of offenders or offenses during 2006–2007.

The data for offenders provided by YRPD contained data on juvenile delinquents, recorded from 1st January 2006 to 31st December 2007. Although the Youth Criminal Justice Act is applicable for juveniles aged between 12 and 17 years (Department of Justice 2017), this study employed data on young delinquents aged between 10 and 19 years old. This age group was used in order to capture the most representative population group of young people from the 2006 Canadian Census. Both males and females involved in at least one offense were counted once in the database (Department of Justice 2017). The offenders were predominantly males and consequently, a separate analysis of males and females was not possible due to the extremely small number of female offenders in some of the DAs, which caused concern of data breach. This small count of female offenders would have been particularly problematic for the 116 of 132 rural DAs, where less than six offenders were found during the study period. Thus, the total count of male and female offenders for all crimes at the DA level, which ranges from 0 to 35, was used for analysis in this study. The offenses data provided by YRPD cover all crimes during 2006 and 2007 that were classified as violent (Uniform Crime Reporting Survey code between 1000 and 2000). They include murder, general and sexual assault, abduction, robbery, and arson, among others. In this data example, we do not have information about the age of offender(s) for each violent crime, and so we confine our analysis to all violent crimes and offenders aged between 10 and 19 years. Below, we will use the terms offenders and offenses when referring to our proposed offenders-offenses model or, in general, but JD and VC when referring to our specific dataset.

**Fig. 1:**
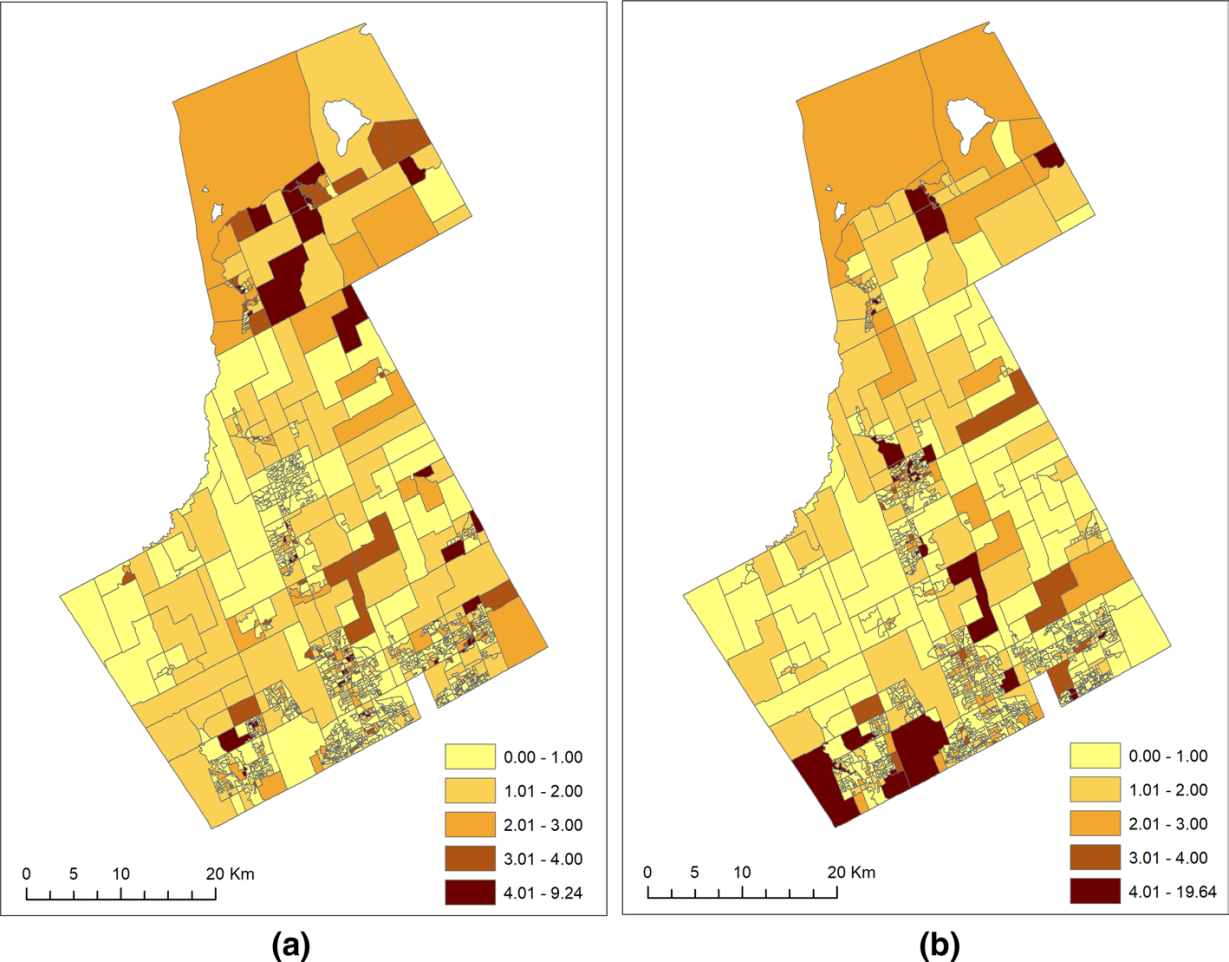
The standardized ratio (crude relative risk) maps of **a** JD and **b** VC

In order to explore the interesting interplay between the offenders and offenses in the study area, we calculated and mapped the area-specific standardized ratios (SR) of the JD and VC separately. This exploratory analysis should not be considered as any model assumption but rather the motivation to adopt a more sophisticated modeling technique such as the SCSM for hotspot detection. The observed and expected counts of the offenders or offenses in each DA were used to calculate these ratios, also referred to as the crude relative risks. The expected count of offenses was calculated using indirect standardization and based on the average trend (from 2006 to 2007) of offenses in the entire York Region multiplied by the residential population (Census 2006) in each DA. The expected count of the JD was also calculated using the indirect standardization method and involved standardizing by sex and age groups of 10–14 and 15–19. Functionally, these expected counts for the offenses represent the level of crime that would be expected if these offenses were distributed proportionally to the residential population. In the case of offenders, the expected counts represent the number of offenders that would be expected in each DA if this number was proportional to each DA's population. Figure 1 maps the SR for offenders and offenses.

A visual interpretation of the SR maps in Fig. 1 shows that the risks owing to the JD and VC are not similar (highest risks for VC), and there are considerable geographic variations in the level of risk. For example, the northern part of the study region exhibits a higher level of SR for JD than for VC. The number of DAs with SR values > 4.00 is nearly twice for JD (n = 42) than for VC (n = 30). Similarly, the DAs that had SR values below 1.00 for VC had SR values higher than 1.00 for JD in the northern portion of the study area. In contrast, the southwestern part of the study region had greater risks (> 4.00) owing to VC than the JD. A similar pattern could be observed in the central portion of the study region, where the presence of DAs with SR values greater than > 4.00 for VC is much more prominent than JD. However, there are more DAs with risks higher than 1.00 for the JD in both the southwestern and southeastern sides compared to the VC in the same area. Exploring these patterns further reiterates the importance of conceptualizing risk surfaces of offenders and offenses through an offenders-offenses shared and offenders- or offenses-specific perspective.

### Shared Component Spatial Modeling (SCSM)

We fitted the shared component model (Held et al. 2005, Knorr‐Held and Best 2001) to examine the joint spatial distributions of offenders (JD) and offenses (VC). Our adopted approach, as proposed by Held et al. (2005), is an extension of the BYM model, which adjusts for the spatial autocorrelation and overdispersion in the count data via the integration of spatially structured and unstructured random effects (Best and Hansell 2009; Haining et al. 2009; Held et al. 2005; Besag et al. 1991). This allows the model to separate the area-specific risks of crime into three components:

**Table:** 

1	Offenders-offenses-shared	(risks shared by both offenders and offenses)
2	Offenders-specific	(risks specific to offenders only)
3	Offenses-specific	(risks specific to offenses only)

In Eq. (1), $O_{ik}$ represents the observed counts in the *i*th area, and *k* is the number of individual components. In this study, *i* = 1, 2, … n, where n is the total number of DAs (n = 1,128), and *k* = 1 or 2, where *k* = 1 denotes the offenders and *k* = 2 denotes the offenses. In other words, $O_{i1}$ is the observed number of offenders and $O_{i2}$ is the observed number of offenses, in an area *i,* during the study period. The observed counts in each area are assumed to follow a Poisson distribution, being conditioned on means $\mu_{i1}$ and $\mu_{i2}$, respectively.

1 $$O_{ik} \sim Poisson \left( {\uplambda_{ik} } \right)$$

2 $$\uplambda_{ik } = e_{ik} r_{ik}$$

Applying logarithm on both sides,

3 $$\log \left( {\uplambda_{ik} |\mu_{ik } } \right) = \log \left( {e_{ik} } \right) + \log \left( {r_{ik} } \right)$$

where the $e_{ik}$ and ${\text{r}}_{ik}$ are the expected count and relative risk, respectively, of *k* for the area *i*.

The SCSM model assumes that offenders and offenses share a similar spatially structured pattern of risk in area *i*, denoted by $\theta_{i}$, referred to as offenders-offenses shared risk. The $\theta_{i}$ term denotes the latent (shared) component of the spatial pattern of risks, as shared by both offenders and offenses in area *i*. The shared component acts as a surrogate for unobserved factors such as deprivation, unemployment, lone parent, and rental housing that may explain the geographical variations of the risk from both offenders and offenses. Risks that are not shared (i.e., not captured by $\theta_{i}$) are captured by the two random effects that follow the BYM model: one is spatially structured, denoted by $s_{ik} ,$ and the other one is unstructured, denoted by $u_{ik}$. These non-shared risks are offenders or offenses specific, where *k* = 1 is for the offenders, and *k* = 2 is for the offenses. Based on these three components ($\theta_{i} , s_{ik} {\text{ and }} u_{ik}$), ${\text{r}}_{ik}$ can be rewritten separately for the offenders (${\text{r}}_{i1} )$ and offenses (${\text{r}}_{i2} )$:

4 $${\text{r}}_{i1} = \exp \left( {\alpha_{1} + \delta \theta_{i} + s_{i1} + u_{i1} } \right)$$

5 $${\text{r}}_{i2} = \exp \left( {\alpha_{2} + \frac{1}{\delta }\theta_{i} + s_{i2} + u_{i2} } \right)$$

Equations (4) and (5) can be used to derive the final model equations from Eq. (3):

6 $$\log \left( {\uplambda_{i1} |\mu_{i1 } } \right) = \log \left( {e_{i1} } \right) + \alpha_{1} + \delta \theta_{i} + s_{i1} + u_{i1}$$

7 $$\log \left( {\uplambda_{i2} |\mu_{i2 } } \right) = \log \left( {e_{i2} } \right) + \alpha_{2} + \frac{1}{\delta }\theta_{i} + s_{i2} + u_{i2}$$

The sum of $s_{ik}$ and $u_{ik}$ is used to capture the type-specific spatial ($s_{ik}$) and non-spatial ($u_{ik}$) patterns that depart from the offenders-offenses shared pattern and represents the latent processes associated with only offenders Eq. (6) or only offenses Eq. (7). The risks due to the between-area spatial autocorrelation and the within-area non-spatial processes are accounted for by the offenders or offenses specific spatially structured ($s_{ik}$) and unstructured ($u_{ik}$) random-effects terms, respectively (Besag et al. 1991). The $\alpha_{1}$ and $\alpha_{2}$ are the specific intercepts for the offenders and offenses, respectively, and represent the baseline risks or the average offenders or offenses risks in an area.

Equations (6) and (7) assume that the offenders and offenses share a common spatial pattern of risk. A scaling parameter $\delta$ ($\delta$ > 0) is used to model the unique association of the offenders or offenses with the shared component $\theta_{i}$ (Lawson 2013). This scaling parameter is required for identifiability and allows different risk gradients to exist for the shared component, which in turn, is produced by the different levels of association between the offenders or offenses with the offenders-offenses shared risk. The model identifiability is enhanced when the scaling parameter $\left( \delta \right)$ of one outcome variable is the inverse of the scaling parameter of the other variable (Lawson 2009, Knorr‐Held and Best 2001). Consequently, we have assigned the offenders with a scaling parameter $\delta$ and the offenses with a scaling parameter $\frac{1}{\delta }$.

A value of $\delta$ close to one shows that the offenders and offenses have a similar level of association with the offenders-offenses shared pattern (i.e., if $\delta = 1$, then $\frac{1}{\delta } = 1$), whereas a large positive value of *δ* indicates that the offenders have a stronger association with the offenders-offenses shared pattern (e.g., if δ = 2, then 1/δ = 0.5). When the ${\text{exp}}(\theta_{i} )$ for an area is greater than 1 at the 95% credible interval (CI), it indicates a high risk due to the offenders-offenses shared pattern in the area. In contrast, when the exp $\left( {\theta_{i} } \right)$ is less than 1 at the 95% CI, the risk due to the offenders-offenses shared pattern is low. Similarly, as the risks due to the offenders or offenses-specific spatial patterns are given by exp $\left( {s_{ik} + u_{ik} } \right)$, areas with high risk due to offenders or offenses will have estimates of exp $\left( {s_{ik} + u_{ik} } \right)$ greater than 1, and areas with low risk due to offenders or offenses will have estimates of exp $\left( {s_{ik} + u_{ik} } \right)$ less than 1.

We assumed non-informative prior distributions where appropriate. For the intercepts, $\alpha_{1}$ and $\alpha_{2}$, we used flat normal priors. For the spatially structured random effects, $\theta_{i} , s_{i1} {\text{ and }} s_{i2} ,$ we assumed an intrinsic normal conditional autoregressive (ICAR) prior distribution with unit weight for neighboring areas that share one or more common boundary points (Law and Haining 2004; Besag et al. 1991). The conditional distribution of $\theta_{i}$ given $\theta_{j}$ can be described as follows:

8 $$\theta_{i} |\theta_{j} , j \ne i, j {\text{ is}}\;{\text{a}}\;{\text{neighbor}}\;{\text{of}}\;i \sim N \left( {\overline{\theta }_{i} , \frac{{\omega_{\theta }^{2} }}{{m_{i} }}} \right)$$

Equation (8) shows that the conditional distribution of $\theta_{i}$ given $\theta_{j}$ is a normal with mean $\overline{\theta }_{i} = \sum\nolimits_{ j \ne i} {\omega_{i,j} \theta_{j} / m_{i} }$ and variance $\frac{{\omega_{\theta }^{2} }}{{m_{i} }}$. The $\omega_{i,j} :i,j = 1,2, \ldots , n$ is a 0–1 contiguity matrix (**W**) in which, $\omega_{i,j} = 1$ when *i* and *j* are neighbors and $\omega_{i,j} = 0$ otherwise, and $\omega_{i,i} = 0$. The $m_{i}$ represents the number of neighbors of the area *i* and can be expressed as $m_{i} = \sum\nolimits_{j} {\omega_{i,j} }$. A similar conditional distribution is applicable for the spatial random effects terms $s_{i1} \;{\text{and}}\;s_{i2}$. The conditional mean of $s_{ik}$ is equal to the mean of the neighboring $s_{jk}$ and have a variance of $\frac{{\sigma_{k}^{2} }}{{m_{i} }}$ (Law and Haining 2004; Law and Perlman 2018).

For hyperprior distributions, we adopted those recommended by Ancelet et al. (2012) following their testing on sensitivity analysis to prior choice for shared spatial-component modeling. For conjugacy and identifiability reasons, the priors recommended for the precision parameters of $\theta_{i} ,s_{i1} , \;{\text{and}}\;s_{i2}$ are all Gamma (0.1, 0.1) and for the unstructured random effects terms ($u_{i1} \;{\text{and}}\;u_{i2}$), a normal prior distribution with mean zero and a precision having prior distribution of Gamma (0.01, 0.01) (Ancelet et al. 2012). A normal prior with a mean of 0 and a precision of 5.9 (variance = 0.17) was used for the $\log \left( \delta \right)$ parameter. This prior is based on the assumption that both $\delta$ and $\frac{1}{\delta }$ are positive and the ratio of the risk gradients ($\delta$ and $\frac{1}{\delta }$) lies between 1/5 and 5 with a 95% probability (Knorr‐Held and Best 2001). For sensitivity analysis, we used uniform priors on [0, 10] for standard deviations of $\theta_{i} ,s_{i1} , \;{\text{and}}\;s_{i2}$ and [0, 100] for standard deviations of $u_{i1} \;{\text{and}}\;u_{i2}$ (Ancelet et al. 2012). It has been acknowledged that there may be some sensitivity to the choice of prior distributions (Held et al. 2005). However, studies on shared spatial-component modeling have shown that the choice of priors had no effect on their main results (Ancelet et al. 2012; Ibáñez-Beroiz et al. 2011).

The shared component model was fitted using WinBUGS (Lunn et al. 2009). It was run to convergence using two Markov Chain Monte Carlo (MCMC) chains. The MCMC chains were initiated at dispersed starting values, and the convergence of model parameters was monitored by trace plots and the Brooks-Gelman-Rubin statistics (Gelman and Rubin 1992) of all relevant parameters. Further samples were taken after convergence to obtain the results. In order to ensure that the number of further samples taken was sufficient, we checked that for each parameter of interest, its Monte Carlo error was less than 5% of the sample posterior standard deviation. These further samples were used to generate the posterior distributions from which estimates of the parameters were obtained by computing the posterior means using the Monte Carlo integration.

### Bayesian Hotspot Identification

The precise meaning of hotspots varies, but in general, hotspots are referred to as areas of high risk. Although several hotspot detection techniques exist for offenders and offenses, such as kernel density estimation, local Moran's *I* and space–time scan statistics; the Bayesian shared-component model can account for uncertainties in the area-specific risk estimates during the hotspot identification process. Furthermore, since in a Bayesian platform, the values of the unknown parameters such as $\alpha_{k}$, $s_{ik}$, $u_{ik}$, $\delta$ and $\theta_{i}$ are estimated from probability distributions, it is possible to carry out probability mapping and identify areas where the model parameters are greater than a researcher-specified threshold (Law et al. 2020). This is known as the *posterior probability mapping* and gives an estimate of the posterior relative risk of the target parameter (Richardson et al. 2004).

In this study, the three components ($\theta_{i} , s_{ik} \;{\text{and}}\;u_{ik}$) of the area-specific risks of crime were used to identify hotspots. More specifically, the posterior probability of each of these components was used for hotspot identification. Offenders-offenses hotspots were evaluated based on the posterior probability of the offenders-offenses shared risk being greater than one (Pr(exp($\theta_{i}$) > 1 | data)). Offenders-specific hotspots were evaluated based on the posterior probability of the offenders-specific risk being greater than one (Pr(exp($s_{i1} + u_{i1}$) > 1 | data)). Likewise, offenses-specific hotspots were evaluated based on the posterior probability of the offenses-specific risk being greater than one (Pr(exp($s_{i2} + u_{i2}$) > 1 | data)) (Law et al. 2015, 2014).

To understand the relative importance of the spatial (shared and specific) and non-spatial processes for analyzing hotspots, we also analyzed the fraction of the total variation in log-relative risk for offenders and offenses explained by the shared component. The results of individual empirical variances were used to calculate these fractions. The same fractions were also directly modeled in WinBUGS to generate the posterior means with credible intervals. Computationally, both the processes are identical and give values of the same outcome, except during the computation of the posterior mean in WinBUGS, the variances of the type-specific components are modeled as ${\text{Var}}\left( {s_{i1} + u_{i1} } \right)$ instead of ${\text{Var}}\left( {s_{i1} } \right) + {\text{Var}}\left( {u_{i1} } \right).$ For offenders, the proportion of variability attributable to the shared component (i.e., fraction shared) equals to ${\text{Var}} \left( {\delta \theta_{i} } \right)/ \left( {{\text{Var }}\left( {\delta \theta_{i} } \right) + {\text{Var}}\left( {s_{i1} } \right) + {\text{Var}}\left( {u_{i1} } \right)} \right)$, which is equal to the empirical variance of $\delta \theta_{i}$ divided by the sum of the empirical variances of $\delta \theta_{i} ,s_{i1} \;{\text{and}}\;u_{i1}$. Similarly, for the offenses, the proportion of variability attributable to the shared component was computed by or ${\text{Var}} \left( {\frac{1}{\delta }\theta_{i} } \right)/ \left( {{\text{Var}} \left( {\frac{1}{\delta }\theta_{i} } \right) + {\text{Var}}\left( {s_{i2} } \right) + {\text{Var}}\left( {u_{i2} } \right)} \right)$, which is equal to the empirical variance of $\frac{1}{\delta }\theta_{i}$ divided by the sum of the empirical variances of $\frac{1}{\delta }\theta_{i}$, $s_{i2}$ and $u_{i2}$.

Finally, we analyzed the spatial pattern of the specific components that were not shared, i.e., specific to offenders or offenses. For offenders, the variance of the spatially structured offenders-specific random-effects terms pattern, which was not shared with offenses, was computed by the empirical variance of $s_{i1}$ divided by the sum of the empirical variances of $\delta \theta_{i}$, $s_{i1}$, and $u_{i1}$. Likewise, we calculated the variance of the spatially structured type-specific random-effects terms for offenses by dividing the empirical variance of $s_{i2}$ using the empirical variances of $\frac{1}{\delta }\theta_{i} ,s_{i2} {\text{ and }} u_{i2}$. The unstructured pattern for offenders was computed by the empirical variance of $u_{i1}$ divided by the sum of $\delta \theta_{i}$, $s_{i1}$, and $u_{i1}$ and for offenses by the empirical variance of $u_{i2}$ divided by the sum of $\frac{1}{\delta }\theta_{i} ,s_{i2} \;{\text{and}}\;u_{i2}$.

## Results

We used the SCSM described above to analyze SCSM hotspots of JD (offenders) and VC (offenses). In fitting the shared component spatial model, for each chain, 20,000 iterations with a thinning of 100 were discarded as burn-in, and an additional 10,000 iterations, thinned by 100, were retained for posterior inference. Table 1 reports the results of the model. Sensitivity analysis showed that the different spatial and non-spatial priors gave similar results.

**Table 1:** Results of the JD-VC (offenders-offenses) shared component spatial model from WinBUGS: The posterior means and credible intervals (2.5–97.5%) of the scaling parameters and empirical variances

	Offenders (JD), k = 1	Offenses (VC), k = 2
Scaling parameter	δ = 1.17 (0.79, 1.64)	1/δ = 0.88 (0.60, 1.25)
*Empirical variances*		
Offenders and offenses shared effects: Var($\delta \theta_{i} )$ for k = 1; Var($1/\delta \theta_{i} )$ for k = 2	0.32 (0.10, 0.62)	0.17 (0.06, 0.34)
Offenders or offenses-specific spatially structured random effects: ${\text{Var}}\left( {s_{ik} } \right)$	0.32 (0.03, 0.63)	0.17 (0.02, 0.33)
Offenders or offenses-specific unstructured random effects: ${\text{Var}}\left( {u_{ik} } \right)$	0.02 (0.001, 0.07)	0.36 (0.30, 0.42)
Offenders or offenses-specific effects (spatially structured and unstructured random effects combined): ${\text{Var}}\left( {s_{ik} + u_{ik} } \right)$	0.34 (0.05, 0.66)	0.53 (0.37, 0.68)
The fraction of total variation in relative risks that was explained by the shared component:	0.49 (0.15, 0.91)	0.25 (0.09, 0.46)

### Offenders-Offenses Shared Patterns

The estimated posterior means of the fraction shared were 0.49 (95% CI: 0.15, 0.91) and 0.25 (95% CI: 0.09, 0.46) for JD and VC, respectively. In other words, the shared component representing the offenders-offenses shared spatial pattern captured 49% of the total variation of JD and 25% of the total variation of VC. The fraction for JD (0.49) was higher than the fraction for VC (0.25), which indicates that the shared component explains a greater proportion of the variability in relative risk for JD than VC.

### Offenders and Offenses-Specific Patterns

For JD, the spatially-structured type-specific random effects terms account for 48% (= 0.32/(0.32 + 0.32 + 0.02) × 100) of its total variation. The unstructured type-specific random effects terms account for only 3% (= 0.02/(0.32 + 0.32 + 0.02) × 100)of its total variation.

For VC, the spatially-structured type-specific random effects terms account for 24% (0.17/(0.17 + 0.17 + 0.36) × 100) of its total variation. The unstructured type-specific random effects terms account for 51% (= 0.36/(0.17 + 0.17 + 0.36) × 100) of its total variation.

**Fig. 2:**
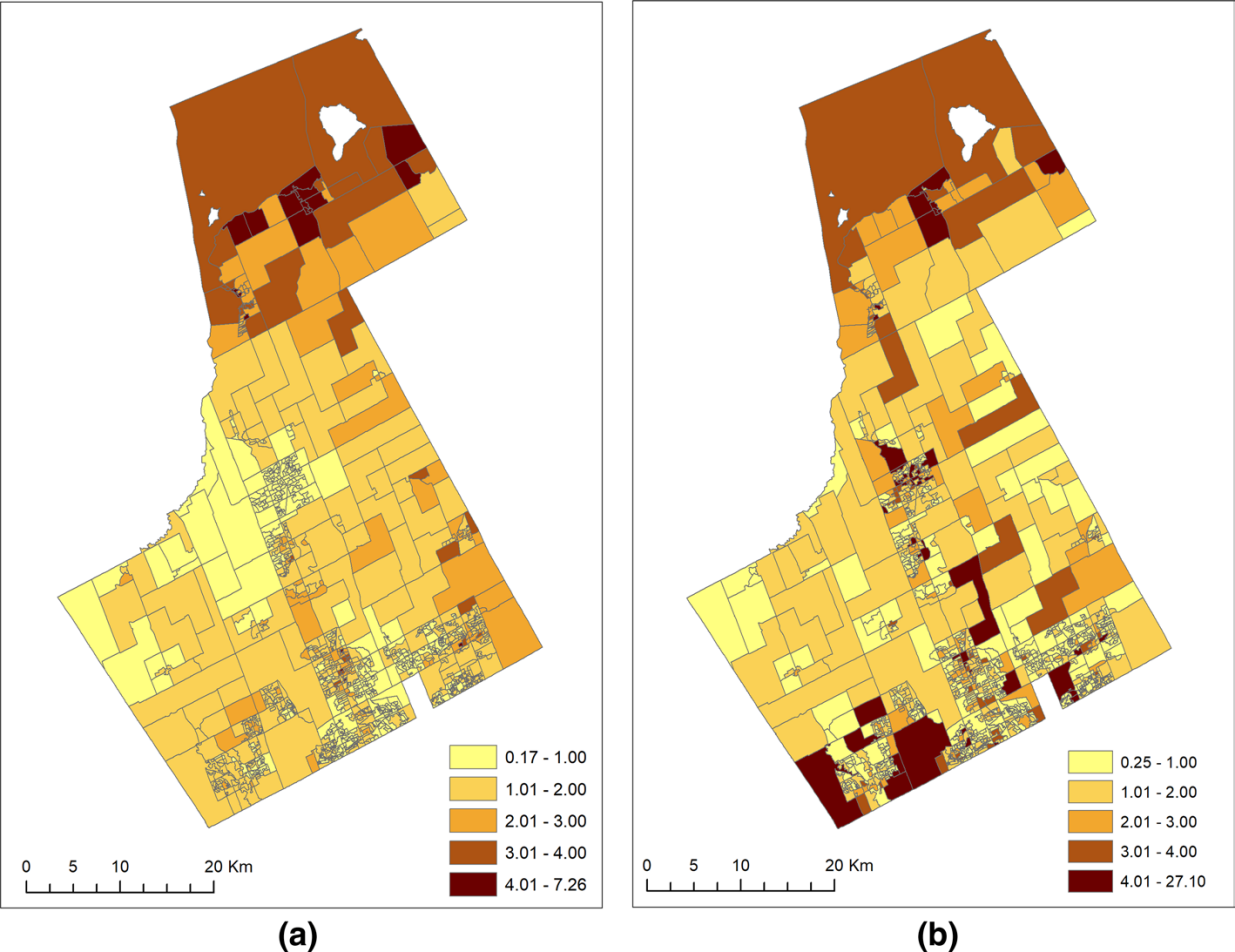
The spatial patterns of relative risks (shared and specific combined) of **a** JD (exp $\left( {\alpha_{1} + \delta \theta_{i} + s_{i1} + u_{i1} } \right)$) and **b** VC (exp $( {\alpha_{2} + \frac{1}{\delta }\theta_{i} + s_{i2} + u_{i2} })$)

Figure 2a and b summarize the variations of (shared and specific combined) risks for JD (offenders) and VC (offenses) from offenders-offenses SCSM by plotting the posterior means of the area-specific risks for JD and VC (Eqs. (4) and (5) above), respectively.

### SCSM Hotspots Mapping

**Fig. 3:**
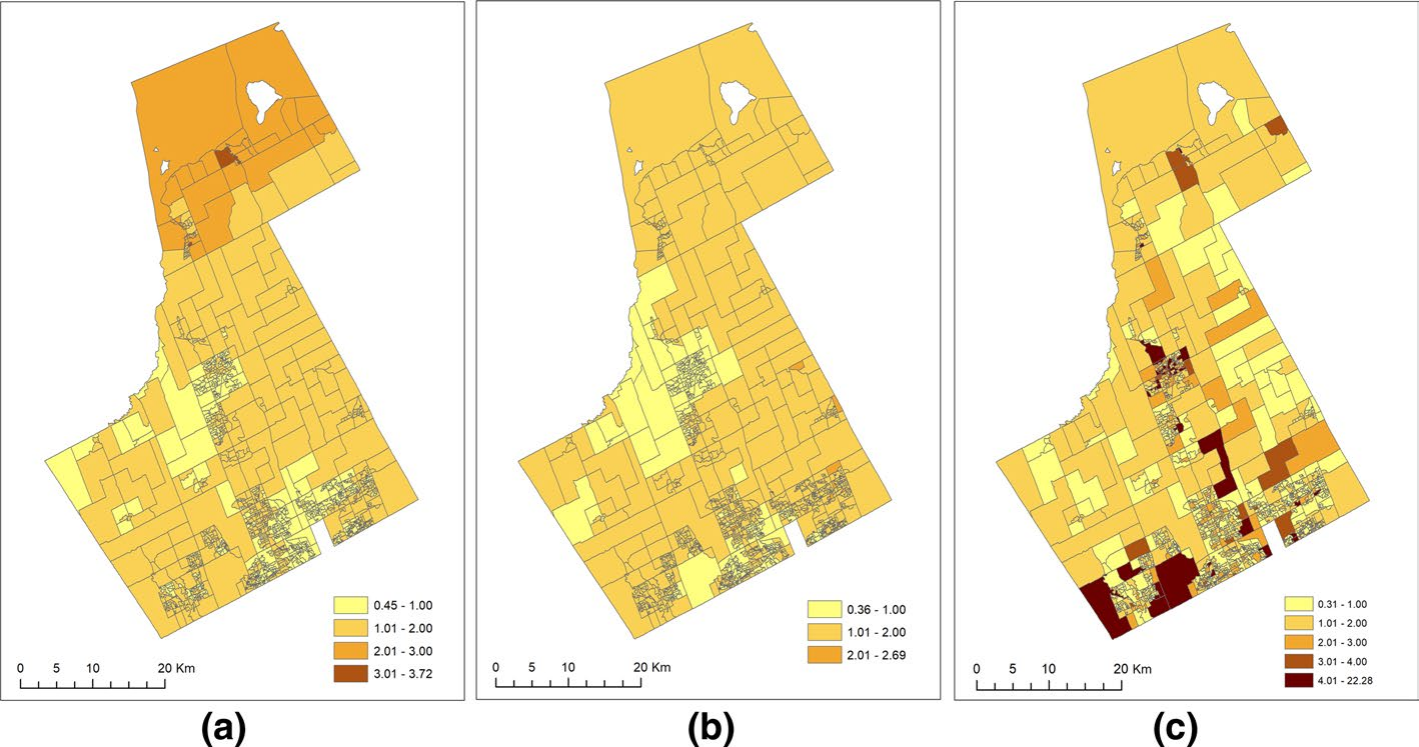
The spatial patterns of **a** JD-VC shared (exp($\theta_{i}$)), **b** JD-specific (exp($s_{i1} + u_{i1}$)) and** c** VC-specific risks (exp($s_{i2} + u_{i2}$))

The results of SCSM enable the identification of hotspots from more than one (offenders and offenses) instead of only one (offenders or offenses) outcome variable. We show below how the spatial variations observed in offenders-offenses shared, offenders-specific, and offenses-specific patterns from the two outcome variables of offenders and offenses (JD and VC in this case study) could be better understood using risk and hotspot mapping. The posterior means for the specific relative risks ranged from 0.36 to 2.69 for JD (Fig. 3b) and between 0.31 and 22.28 for VC (Fig. 3c). The type-specific risk map (Fig. 3b) for JD shows that the variation of specific-risk for the JD was not large throughout the study region. Relatively high risk (> 1.00) could be observed in the entire northern, eastern, and southern regions. However, for the VC (Fig. 3c), the risk pattern was similar to a checkerboard pattern with a risk greater than 4 in many areas. A varying level of risks was evident in the northern, central, eastern, and southern regions. Therefore, the risks due to VC (offenses-specific) were more scattered throughout the region and did not conform to any distinct spatial pattern.

Figure 3a displays the map for the posterior means of the unscaled shared relative risk. The posterior means shared relative risks ranged from 0.45 to 3.72. The map represents the dissemination area level spatially structured variability common to JD and VC. It can be interpreted as the underlying risk surface when considering both JD and VC in the study region. We observed that this shared relative risk map is quite identical to the JD estimated risk map (Fig. 2a) except for the northern part of the study area, where the shared relative risk was relatively lower compared to the JD risk.

**Fig. 4:**
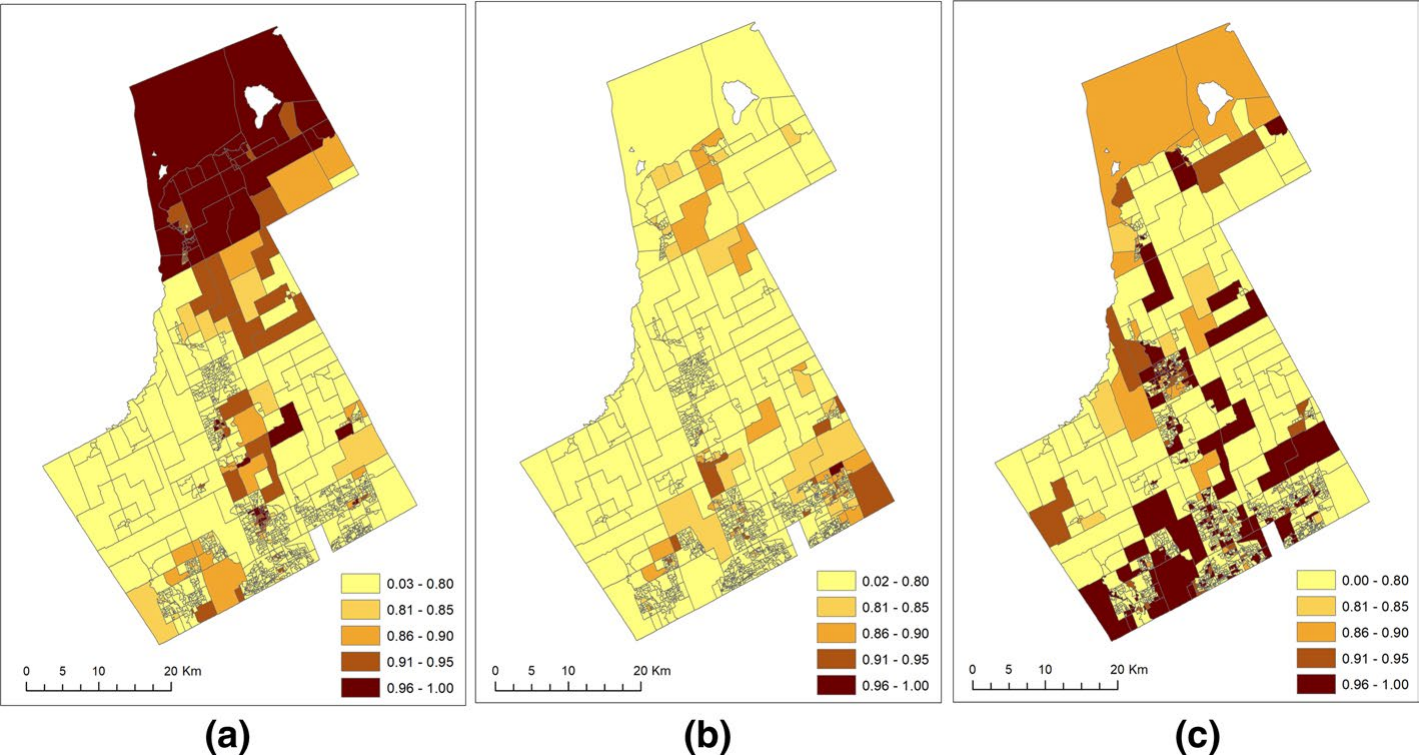
The hotspots (posterior probability of risk greater than one) for **a** JD-VC shared (Pr(exp($\theta_{i}$) > 1 | data)), **b** JD-specific (Pr(exp($s_{i1} + u_{i1}$) > 1 | data)), and **c** VC-specific hotspots (Pr(exp($s_{i2} + u_{i2}$) > 1 | data))

We use hotspot maps to illustrate the estimated posterior probability of offenders-offenses shared (Pr(exp($\theta_{i}$) > 1 | data)), and offenders or offenses-specific risks that were greater than 1 (Pr(exp($s_{ik} + u_{ik}$) > 1 | data). Figure 4b reveals that there were clusters of areas with a high probability of specific risks greater than 1 in the northcentral and southeastern portion of the study area. Despite having similar type-specific risk levels (1.01 < exp($s_{i1} + u_{i1}$) < 2.00), the variations in the posterior probability revealed distinct spatial patterns between the northcentral and southeastern parts. However, we can see from the VC-specific probability map in Fig. 4c that the high probability (0.96–1.00) of risks greater than one of VC was dispersed throughout the study region. The checkerboard pattern of the type-specific risk for VC became more prominent in the hotspot map when compared with the relative risk maps. In contrast, large clusters of JD and VC-shared hotspots (Fig. 4a) could be observed north of the study area.

## Discussion

Despite the strong interdependence suggested by the ecological and social theories of crime, studies analyzing the shared risk of offenders and offenses are rare. This study has attempted to fill this void and introduced a Bayesian SCSM approach, where the offenders (juvenile delinquents) and offenses (violent crimes) are jointly studied using a single statistical model. Since offenders and offenses are considered separate entities by the model, both the shared and specific risks could be estimated to map the offenders-specific, offenses-specific, and offenders-offenses shared hotspots. We discuss below the use of SCSM for identifying hotspots, its potential for other applications such as identifying associations, limitations of our study, and future research.

### Interpretations of the Results

The Bayesian SCSM approach presented in this study highlights some crucial aspects of managing offenders and offenses as such in the case of juvenile delinquency and violent crimes in an area. Due to resource scarcity in large administrative regions, it is not possible to simply increase patrolling in the high-risk areas of JD or VC (Law and Haining 2004). Thus, control strategies targeting JD-VC shared hotspots could be undertaken on a priority basis, as in doing so, both the JD and VC could be controlled simultaneously (Law et al. 2015, 2020). In this regard, offenders-offenses shared hotspots hold considerable operational significance as it allows multiple aspects of crime to be targeted simultaneously.

The scaling parameter (δ) in the SCSM estimates the difference between the magnitudes of JD and VC area-specific risks. Our results of the posterior means of the scaling parameter δ and 1/δ equal 1.17 (95% CI: 0.79, 1.64) and 0.88 (95% CI: 0.60, 1.25), respectively. The 95% CI for δ includes the value 1, indicating that the study region has similar risk gradients of JD and VC. That is to say, JD and VC have a similar level of association with the JD-VC shared pattern. The result provides no evidence that the contributions to the shared component by JD and VC differed. As expected, the ratio of the risk gradients of JD and VC equals 1.32 (= 1.17/0.88) and is between 1/5 and 5. The ratio suggests that the shared component was playing a more dominant role in the area-specific risks of JD than those of VC. The fraction of total variation in relative risks explained by the shared component for JD equals 0.49, which indicates that nearly 50% of the variability of JD is attributable to the shared component (of offenders and offenses). Similarly, the fraction of 0.25 for VC shows that 25% of the variability of VC is due to the shared component. So, targeting those areas with high risks on the shared component map would mean targeting 50% of JD that is location-wise common to VC or 25% of VC that is location-wise common to JD in the study region.

Additionally, the use of SCSM allows the spatially structured ($s_{ik}$) and unstructured ($u_{ik}$) random effects to be considered as individual model components. It enables us to understand whether the spatially structured (unknown) covariates influence the distribution of JD and VC more than the unstructured ones. For example, we found that the spatially structured covariates caused 48% of the total variations of the JD. Compared to this, only 24% of the total variations of the VC were caused by the spatially structured covariates. The two percentages indicate that the distribution of JD after accounting for shared risks had a more prominent spatial pattern compared to VC. This contrast can also be seen when comparing the type-specific (i.e., excluding the shared component) risk maps in Fig. 3b and c and the type-specific hotspots in Fig. 4b and c for JD and VC, respectively. We can also see from Fig. 4b that JD has a less unstructured or random pattern than VC (Fig. 4c), which explains why the unstructured random effects account for a lesser amount of the total variation for JD than VC (3% versus 51%).

### Conceptualizing the Relationship Between Offenders and Offenses Using Bayesian SCSM

The Bayesian SCSM approach proposed by this study has shown several specific methodological advantages in understanding the relationship between offenders and offenses, which were not found in past studies. Specifically, through the integration of the shared component (denoted by the $\theta_{i}$ term) of the spatial pattern of risks, SCSM allows researchers to estimate the degree to which offenders and offenses could be influenced by targeting the areas with high risks on the shared component (the offenders-offenses hotspots, see Fig. 4a).

We conceptualized the entire study area as having some degree of risk from the offender and offenses and further conceive the results to be providing estimates of three risk surfaces: (1) shared by both offenders and offenses (Fig. 3a), (2) specific for offenders (Fig. 3b), and (3) specific for offenses (Fig. 3c). Then, we extended the risk-surface analysis to hotspot mapping by estimating and mapping the probability of the aforementioned three types of risk that is greater than 1. The hotspot maps (Fig. 4) were then used to identify hotspots. (i.e., based on the posterior probability of risk > 1) owing to both offenders and offenses (shared hotspots, Fig. 4a), and offenders (Fig. 4b) or offenses (Fig. 4c) only. The concept of shared hotspots aligns with the work of Normandeau (1968), as the location of shared patterns is assumed to be the locations where the offenders and victims co-exist or meet at the same location for the offense to take place. Consequently, these areas with high probabilities of shared risks have both offenders and offenses problems, suggesting issues that conjointly affect the distribution of offenders and offenses and possibly an association between them in those areas. As a result, targeting offenses in the shared hotspots (Fig. 4a) could also help decrease the number of offenders in the long run. However, it is important to note that the offenders and offenses data should correspond to the same crime type(s) before any final conclusion could be made on their associations or targeting issues that are common to both.

After accounting for the shared risks (Fig. 4a), the offenders-specific (Fig. 4b) and offenses-specific (Fig. 4c) risks were used to highlight areas that have a high posterior probability of risks > 1 due to offenders and offenses, respectively. These areas could have societal issues (such as poor neighborhood conditions) that impact people to offend or spatial attributes (such as poorly lit roads) enabling offenders to offend. Thus, filtering out the offenders-specific risk surface from that of the offenses-specific surface helps probabilistic hotspot mapping and targeted search for risk factors that determine the distributions of offenders and offenses in space. Although past studies attempted to detect the offenders-specific or the offenses-specific hotspots (Bowers et al. 2004; Malleson and Andresen 2015; Pope and Song 2015), the major difference between this study and the past studies lie in analyzing both the crime-shared and crime-specific (offenders-specific and offenses-specific) hotspots together in the same model. Therefore, in principle and due to the application of SCSM, the offenders-specific and the offenses-specific hotspots detection technique proposed in this study are produced by evaluating the risk from offenders or offenses only, after filtering out the spurious offenders-offenses shared risk.

From another perspective, to interpret the results from SCSM, we relate the offenders-offenses shared and specific hotspots identified to the mobility triangle in terms of offenders and offenses at the DA (small-area) level. The offenders-offenses shared hotspots could depict the locations of DA triangles (offenders, offenses, and victims all located in the same DA, as observed for the cases of domestic violence) and victim mobility triangles (offenders and offenses same DA, but victims elsewhere, e.g., robbery). In contrast, the specific (offenders or offenses) hotspots could depict the locations of the *offender mobility triangle* (offenses and victims same DA but offenders elsewhere, e.g., arson), *offense mobility triangle* (offender and victim same DA, but offense elsewhere, e.g., murder), and *total mobility triangles* (offender, offenses, and victims all from different DAs, e.g., abduction). Thus, the proposed SCSM technique for detecting hotspots can take into account the intricate relationship typology between the offenders, victims, and offenses (Normandeau 1968; Groff and McEwen 2007; Tita and Griffiths 2005). Although our work did not analyze the victim-specific hotspots for simplicity and data scarcity reasons, the proposed multivariate SCSM technique could be extended to incorporate an additional victim-specific outcome. Doing so would help identify the offenders-victims-offenses shared hotspots and type-specific hotspots for offenders, victims, and offenses.

Furthermore, we observed that the variance of shared spatial risk for JD has a value of 0.32 (95% CI: 0.10, 0.62) and for VC 0.17 (95% CI: 0.06, 0.34). These values suggest that the offenders (JD) could show a relatively larger variation for the shared risk than the offenses (VC). A similar finding could be observed when the variances of the type-specific spatial risk $\left( {s_{ik} } \right)$ for JD (0.32; 95% CI: 0.03, 0.63) and VC (0.17; 95% CI: 0.02, 0.33) are compared, which suggests similarities in the spatial pattern for shared and offender-specific risks. This is concordant with our past studies and findings where we observed that offenses (compared to the result of variation of spatial risk of offenders in this study) show a relatively smaller variation for the shared and type-specific spatial risks (Quick et al. 2019; Law et al. 2020). However, after conjointly studying JD and VC in the same multivariate model in this study, we obtained this interesting finding that the spatial risk (both shared and type-specific risk) of JD is higher when considered in conjunction with the risk from VC. The mobility triangle can explain this relatively larger spatial variation of the shared and offender-specific risk. In reality, offenders could be more mobile than the offenses, and the same offender may move to different places to find a victim or commit an offense where conditions are favorable for crimes and for attracting offenders from many areas. Compared to this, the offenses (committed by the offenders) are relatively less mobile, and some offenses (e.g., property crimes) could be localized in only certain areas (e.g., rich neighborhoods).

In theory, crime could be controlled by targeting any one of the mobility triangle's arms. For example, for the offender mobility triangle, preventing offenders from reaching the site of the offenses and victims helps prevent arson. Similarly, for the offense mobility triangle, preventing either the offender or victim from reaching the offense site could help prevent the murder. However, simultaneously targeting two arms produces better results in crime management practices. For instance, preventing offenders from reaching the site of offenses and, at the same time, strict monitoring of the site of offense renders it unsuitable for the crime. This ultimately helps an effective reduction of crime. Through SCSM, it is possible to visualize and understand these mobility triangles better and undertake targeted interventions.

Although the SCSM did not explicitly incorporate potential risk factors, conceptually, the shared component still captured the latent risk factors that were simultaneously associated with offenders and offenses (Quick et al. 2019; MacNab 2010; Held et al. 2005). Our results of shared hotspots suggest that JD and VC had a shared component, which suggests that JD and VC had common risk factors and that JD quite often lived in areas that had VC. Since the $\theta_{i}$ term denotes the latent (shared) component of the spatial pattern of risks shared by both offenders and offenses, it acts as a surrogate for any unobserved factors that may explain the geographical variations of the risk of offenders and offenses. An area (*i*) with a high risk of both offenders and offenses is indicated by a large positive value of $\theta_{i}$. Such areas are common in Fig. 3a, suggesting an association between JD and VC or the two outcomes being modeled by the SCSM in this study. Our ongoing research will investigate the use of SCSM for identifying associations and shared and specific hotspots that remain after controlling for known risk factors. It will compare the performance of SCSM and spatial regression analyses to identify associations between the two components.

### Limitations and Scope for Further Improvements

A further modification to our proposed SCSM model has been explored, by which the spatiotemporal association of different putative risk factors with offenders and offenses could be analyzed using a single model (Quick et al. 2019). We believe that a suite of short-term and long-term intervention strategies, focusing on the spatial and non-spatial risk factors and the offenders-offenses and crime-specific hotspots, has the potential to revolutionize crime management practices in the future.

Despite the strengths of the study, a number of improvements can be made to overcome the limitations and further develop new applications of the SCSM for law enforcement planning. First, we have aggregated different sub-types of violent crimes (committed by all age groups) and juvenile delinquents before inputting them into the model due to data limitations. Studies have shown that the risks might vary based on these sub-types of offenses and offenders (Quick et al. 2019; Izenman et al. 2011). Ideally, the offenders and offenses data should match when using an offenders-offenses model. For instance, it would make better sense for us to use specific VC that were committed by JD only or JD who committed a particular VC only for this study. This implies that for the SCSM model, we would use the datasets for murder (or any other sub-types of VC) and JD who had committed murders. This is an important limitation of our study due to the unavailability of data, which future research could try to overcome using more readily available data. However, since our main objective was to demonstrate a new approach to analyzing offenders and offenses using SCSM, we have aggregated these data to simplify interpretations and focus more on the different components of the SCSM.

Future research may consider analyzing how the shared and specific risks vary across different sub-types of offenders and offenses; however, data of offenders with details of their residence information and specific crime committed are often difficult to acquire due to privacy concerns. Using offenders-offenses shared component analysis with appropriate data may provide insights into the location(s) of the offender(s) for a specific crime or crime type by interpreting the results of shared (offender and offense) or offenders-specific hotspot maps. For instance, if we know that the offender of a robbery was young, then it is possible that the offenders-specific hotspot map (like Fig. 4b) from an offender (JD)- offense (VC or robbery-specific, depending on data availability) analysis can provide some insights into the most probable locations of the offender, assuming that the offender was not living in the neighborhood where the offense occurred. With further research, the offenders-offenses shared component analysis could become a different or helpful approach of geographic profiling (Rossmo 1999) for locating offenders.

We assumed that all offenders and offenses were reported and recorded in our dataset, which might not be the case. Additionally, we assumed that the offenders are stationary when calculating the expected number of offenders. In reality, offenders can be mobile and move from one area to another (Johnson et al. 2009), which could affect the offenders-specific risk surface (Law et al. 2020). Unfortunately, this is a limitation caused by the existing data that does not allow the mobility of the offenders to be captured satisfactorily. With the advent of more sophisticated data collection techniques, future research may explore how this mobility influence risk surface estimation and the detection of offenders-specific and offenders-offenses hotspots.

## Conclusion

Evaluating the risk of a crime involves modeling the complex interactions between offenders and offenses. The spatial distributions of offenders and offenses have long been studied from univariate lenses, which involves considering offenders and offenses as separate entities. In cases where these two were studied using multivariate models, the colocation of offenders and offenses could not be studied due to the limitations of the modeling techniques.

This study, using the data for juvenile delinquents and violent crimes as an example of offenders and offenses, respectively, attempted to overcome the major constraints of studying the risk surfaces of offenders and offenses. The study demonstrated the application of Bayesian shared component spatial modeling (SCSM) and modeled the offenders-offenses shared, offenders-specific and offenses-specific risks. The use of SCSM allowed the joint analysis of offenders and offenses in one statistical model but as two separate outcomes that are analyzed together. The spatially structured and unstructured latent covariates, affecting the risk distribution of juvenile delinquents (JD) and violent crimes (VC), were integrated into the SCSM model by two random effect terms. The posterior probability mapping was used to identify the high-risk areas that are either offenders-offenses shared or offenders-specific or offenses-specific in nature.

Results suggest that the shared component of the SCSM model captured the offenders-offenses shared risk areas that are location-wise common to both the offenders and offenses. These areas hold considerable operational significance, as targeting these areas implies controlling the risks that are common to both. The spatially structured and unstructured random effect terms allowed the mapping of offenders- and offenses-specific hotspots, which in turn, could help shed light on the mobility of offenders and offenses. The hotspot areas could be intricately studied to understand the spatial and non-spatial risk factors influencing both the frequency and distribution of offenders and offenses in the study area.

Our work presented an alternative way of conceptualizing the relationships between offenders and offenses. The future use of SCSM can provide valuable insights into the most probable locations of the offenders and offenses while accounting for putative risk factors.

## Data Availability

The dataset analyzed in this study was provided by the York Regional Police Department, Ontario, Canada. Therefore, data-sharing restrictions are applicable for the analyzed dataset.
